# Unusual Psychosis

**DOI:** 10.1192/bjo.2026.11150

**Published:** 2026-06-30

**Authors:** Doaa Abozaid

**Affiliations:** Derbyshire Healthcare NHS Foundation Trust, Chesterfield, United Kingdom

## Abstract

**Aims:**

Exploring an unusual, interesting case of Postictal Psychosis.

**Methods:**

Gathering data from an encountered admission and previous hospital letters.

**Results:**

An interesting case that I had encountered through the initial 6 months of my training where a 46-year-old male was admitted for 2 years in hospital. He grew up living with his mother and two siblings. His premorbid personality described as extroverted, enjoyed reading books, playing bowls and watching the news. He is known to have trouble with dealing with frustration and coping with stress. He had a diagnosis of refractory epilepsy which limited his career to working in the family off licence shop, and also left school at the age of 16 due to bullying.

His past medical history included:

Severe Refractory Focal Epilepsy with Complex Parietal and Generalised seizures (Epilepsy first diagnosed at 15).

Right Temporal Cavernoma–surgical excision (2001), Hypothyroidism, Acute Myeloid Leukaemia (1986), Splenectomy, Bone Marrow Transplant, Iron Deficiency Anaemia.

He doesn't have any history of use of illicit drugs or alcohol.

He was first diagnosed with epilepsy at the age of 15. In the 2000s (Early) was having frequent seizures, family reported he’s reducing dose of AED on his own. He was admitted in November 2001 with complex partial status that required an ITU stay which led to a marked improvement in his seizure frequency. The week before admission he had become increasingly disturbed and developed psychotic behaviour. He was diagnosed with forced normalisation. He was given flupentixol.

He was trialled on most AEDs and was on 5 AEDs at the same time.

Mental health history included frequent admission under MHA and informally. He had frequent contact with Crisis team and ED and he was reported as a frequent caller. Hereported auditory and visual hallucinations specially after seizures. He was trialled onrisperidone, risperidone depot, haloperidol, lorazepam and diazepam, flupentixol.

He had multiple investigations throughout the years including MRI and CT head which didn't show any acute changes.

He was diagnosed eventually with Postictal Psychosis and Personality disorder according to Logsdail and Toone's criteria.

**Conclusion:**

It is important to note the “herald symptoms” that occur hours before the psychosis sets in. The person becomes restless and irritable. They also usually develop insomnia.

Treatment includes: benzodiazepines, low-dose antipsychotic medication, RNS or deep brain stimulation. Surgery also may be an option, but risk must be calculated properly and the key is preventing seizures.

